# A robust approach based on Weibull distribution for clustering gene expression data

**DOI:** 10.1186/1748-7188-6-14

**Published:** 2011-05-31

**Authors:** Huakun Wang, Zhenzhen Wang, Xia Li, Binsheng Gong, Lixin Feng, Ying Zhou

**Affiliations:** 1 College of Bioinformatics Science and Technology, Harbin Medical University, Harbin, 150081, PR China; 2 School of Mathematical sciences, Heilongjiang University, Harbin, 150080, PR China

## Abstract

**Background:**

Clustering is a widely used technique for analysis of gene expression data. Most clustering methods group genes based on the distances, while few methods group genes according to the similarities of the distributions of the gene expression levels. Furthermore, as the biological annotation resources accumulated, an increasing number of genes have been annotated into functional categories. As a result, evaluating the performance of clustering methods in terms of the functional consistency of the resulting clusters is of great interest.

**Results:**

In this paper, we proposed the WDCM (Weibull Distribution-based Clustering Method), a robust approach for clustering gene expression data, in which the gene expressions of individual genes are considered as the random variables following unique Weibull distributions. Our WDCM is based on the concept that the genes with similar expression profiles have similar distribution parameters, and thus the genes are clustered via the Weibull distribution parameters. We used the WDCM to cluster three cancer gene expression data sets from the lung cancer, B-cell follicular lymphoma and bladder carcinoma and obtained well-clustered results. We compared the performance of WDCM with k-means and Self Organizing Map (SOM) using functional annotation information given by the Gene Ontology (GO). The results showed that the functional annotation ratios of WDCM are higher than those of the other methods. We also utilized the external measure Adjusted Rand Index to validate the performance of the WDCM. The comparative results demonstrate that the WDCM provides the better clustering performance compared to k-means and SOM algorithms. The merit of the proposed WDCM is that it can be applied to cluster incomplete gene expression data without imputing the missing values. Moreover, the robustness of WDCM is also evaluated on the incomplete data sets.

**Conclusions:**

The results demonstrate that our WDCM produces clusters with more consistent functional annotations than the other methods. The WDCM is also verified to be robust and is capable of clustering gene expression data containing a small quantity of missing values.

## Background

The changes of the gene expression levels are very common in the human complex diseases, such as cancers [1-3]. The advent of microarray technologies have made it possible to measure simultaneously the expression levels of many thousands of genes over different time points and/or under different experimental conditions [4-6]. Numerous computational techniques have been developed to analyze these gene expression data. Among them, clustering is a primary approach to group the genes with similar expression patterns across different conditions, which enables the identification of differentially expressed gene sets in cancerous tissues [7-9]. Clustering is an unsupervised learning technique which assigns a set of objects (genes) into subsets (called *clusters*) so that the objects in the same clusters are similar according to some similarity metric [10,11]. A cluster is therefore a collection of objects which are similar between them and are dissimilar to the objects belonging to other clusters.

Since clustering is proposed, an increasing number of clustering approaches have been developed and improved for the analyses of gene expression data. The common clustering methods include k-means [12,13], hierarchical clustering [8], and Self Organizing Map (SOM) [14,15], and so on. Each method has its own strengths and weaknesses. The k-means is an important clustering algorithm which partitions n objects into k clusters in which each object belongs to the cluster with the nearest mean. In k-means clustering, the number of clusters k is an input parameter, and an inappropriate choice of k may yield poor clustering results. The main advantages of this algorithm are its simplicity and computational speed which allows it to run on large datasets, however, it does not yield the same result with each run, since the resulting clusters depend on the initial random assignments. Besides, it conducts poorly with overlapping clusters and is sensitive for noisy data. The hierarchical clustering aims to create a hierarchy of clusters which may be represented by a tree structure called a dendrogram. The root of the tree consists of a single cluster containing all objects, and the leaves correspond to individual objects. The hierarchical technique requires relatively smooth data and the clusters themselves need to be well defined. Like k-means method, noisy data strongly affect the resulting clusters. SOM is a type of artificial neural network that is trained using unsupervised learning to produce a two-dimensional, discretized representation of the input space of observations. It requires the geometry of nodes as input, and the nodes are mapped into two-dimensional space, initially at random, and then iteratively adjusted. SOM imposes the structure on data, with neighboring nodes tending to define related clusters. SOM has good computational properties and is suited to clustering of large data sets. One major drawback of this algorithm is the "boundary effect" of nodes on the edges of the network, which may lead to less effective clustering results. Besides, these clustering methods mentioned above require a complete data set as an input, and therefore those gene rows containing the missing values are either removed or imputed using an imputation method on the missing entries prior to clustering analysis. Removing the missing gene rows may result in omitting some important genes, such as the genes related to diseases, whereas the badly estimated missing values even changes the quality of data, which could influence the accuracy of clustering results.

In this article, we propose a Weibull distribution-based clustering method called WDCM. The assumption of this method is that the gene expression of each gene can be considered as a random variable following unique Weibull distribution [16], and that a group of genes tend to be clustered together if the Weibull distributions of gene expressions of these genes have similar distribution parameters. Here, we use the gene expression values of each gene to construct its corresponding Weibull distribution and then group these genes by clustering their corresponding distribution parameters.

The following sections of this paper are organized as 'Results', 'Discussion and conclusion' and 'Methods'. In section 'Results', we first introduced three cancer gene expression data sets we used, and then visually demonstrated the clustering results obtained using the WDCM for the three data sets. Second, to assess the performance of the WDCM, we compared the functional consistency of the gene clusters produced by the WDCM to those of the k-means and SOM methods for the same data sets. We also used the external measure Adjusted Rand Index to establish the performance of the WDCM, and the comparisons with the other algorithms were conducted simultaneously. Finally, we tested the robustness of the WDCM on clustering the incomplete data sets. In section 'Discussion and conclusion', we first summarized the main work of this study, discussed the strength and limitation of the WDCM. In the end we briefly mentioned the improvement of the WDCM and the future study. In section 'Methods', we introduced the WDCM together with the algorithm used for clustering the Weibull distribution parameters, the functional consistency assessment method of the clustering result, and the external validation index Adjusted Rand Index of the clustering performance. Moreover, Robustness test of the WDCM on clustering the incomplete data set was also presented in this section.

## Methods

In this section, the WDCM is described as follows: Given a m × n gene expression matrix, let *g*_*ij *_be the *jth *expression value of gene *i, i *= 1, ...,*m*, and *j *= 1, ...,*n*. We here treat one gene expression as a random variable, and construct the distribution of the gene expressions of gene *i*. We then choose a subset of genes whose distributions of the gene expressions belong to the common Weibull distribution [16]. Due to the consistent distribution function types, we consider that those genes with similar gene expression distribution parameters tend to share the similar expression patterns, and they are probably concerned with the same biological processes or functions together. We further cluster the genes in the selected subset by clustering their corresponding distribution parameters, as each gene corresponds to its unique distribution parameters. In the following we introduce the principle of the distribution function construction procedures.

### Weibull distributions of gene expressions construction

First, we construct the empirical distribution of each gene expression [17], and then ascertain the precise distribution regarding the constructed empirical distribution using the Kolmogorov goodness of fit test [18-20]. The details as follows: assume that *x*_*i*1_, *x*_*i*2_, ..., *x*_*in *_are the gene expressions of gene *gi, i *= 1, ...,*m*, and sort them in ascending as . For ∀ *x *∈(-∞,+∞), define the empirical distribution of *g*_*i *_as(1)

Where *I*(∙) is the indicator function.

We utilize the Weibull distribution type to fit , and then ascertain the distribution parameters which uniquely determine the distribution.

The probability density function of a Weibull distribution is defined as:(2)

where *a *>0 is the scale parameter and *b *>0 is the shape parameter of the distribution. The scale parameter *a *determines the range of the distribution. The shape parameter *b *is what gives the Weibull distribution its flexibility. By changing the value of the shape parameter, the Weibull distribution can fit a wide variety of data.

Let F^(*i*)^(*x*) is a certain Weibull distribution with known parameters, and a Kolmogorov-Smirnov test is conducted to determine if the sample *x*_*i*1_,*x*_*i*2_, ..., *x*_*in *_comes from the Weibull distribution F^(*i*)^(*x*). The null hypothesis is that the random sample of gene expressions of *g*_*i *_comes from the Weibull distribution F^(*i*)^(*x*). If the null hypothesis is true, the deviation of F^(*i*)^(*x*) and F^(*i*)^(*x*) is small. Construct the Kolmogorov-Smironov statistic(3)

under the null hypothesis,  converges to the Kolmogorov distribution [18]. The null hypothesis is rejected at significance level *α *if , otherwise it is accepted, where *K*_*α *_is the critical value of the Kolmogorov distribution. Given *α *= 0.05, we here select the appropriate parameters for *F*^(*i*)^(*x*) in order to the null hypothesis is accepted (*p *- *value *> 0.05), that is, the random sample comes from the certain Weibull distribution *F*^(*i*)^(*x*), *i *= 1,2, ...,*m*. Following the above procedure, we can obtain the Weibull distributions of *m *gene expressions, denoted by *F*^(1)^(*x*),*F*^(2)^(*x*),...,*F*(*m*)(*x*).

### Weibull distribution parameters of gene expressions clustering

Let *θ*_*i *_denotes the parameter of the Weibull distribution *F*^(*i*)^(*x*), *j *= 1, ...,*m*. Here *θ*_*i *_consists of double-parameter pair (*a*_*i*_,*b*_*i*_), we then cluster the *m *parameters *θ*_1_, *θ*_2_,..., *θ*_*m *_using a certain clustering algorithm based on the hub points. This algorithm presented by Robert Clason designates a single point as a hub for each cluster and then finds the distance from each remaining point to each hub, as well as assigns this point to the hub to which it is closer [21]. The merit of it is to automatically ascertain the clusters number on the basis of the distances between data points. A detailed description of the algorithm is provided in Additional file 1.

### Functional consistency of clustering result

In order to evaluate the performance of the proposed WDCM, we also apply the K-means and Self Organizing Map (SOM) clustering algorithms to the same gene subsets as the WDCM and obtain the gene clusters, respectively. We compare the functional consistency of the gene clusters produced by WDCM to those produced by the other methods. For this purpose, we consider the biological annotations of the gene clusters in terms of Gene Ontology (GO). The Gene Ontology (GO) project provides three structured, controlled vocabularies that describe the gene products in terms of their associated biological processes (BP), cellular components (CC) and molecular functions (MF) [22]. The annotation ratios of each gene cluster in three GO terms were calculated using the web-accessible DAVID 2008 tool [23]. For each of clusters found by one of three clustering methods, under the BP ontology, we search the just GO term in which the most genes in this cluster are enriched, and define the BP annotation ratio for this cluster as the number of genes in both the assigned GO term and this cluster divided by the number of genes in this cluster. After calculating the BP annotation ratios for all clusters, we treat the mean value of all annotation ratios as the final BP annotation ratio. We also define the CC and MF annotation ratios by the same manner. A higher annotation ratio represents that the corresponding clustering result is better than the other ones, that is, gene are better clustered by function, indicating a more functionally consistent clustering result.

### Adjusted Rand Index validation index

The Adjusted Rand Index (*ARI*) is a measure of agreement between two partitions of the same set of objects [24,25]. One partition is given by the clustering method and the other is defined by the external criteria. For a gene expression data set, suppose *X *is the partition based on some external criteria and *C *is the clustering result obtained by some clustering method. Let *a,b,c,d *respectively denote the number of gene pairs that are in the same cluster in both *X *and *C*, the number of gene pairs that are in the same cluster in *X *and in different clusters in *C*, the number of gene pairs that are in different clusters in *X *and in the same cluster in *C *and the number of gene pairs that are in different clusters in both *X *and *C*. The Adjusted Rand Index *ARI*(*X,C*) is defined as follows:(4)

The value of Adjusted Rand Index varies from 0 to 1 and higher value means that *C *is more similar to *X*.

Considering that the genes with similar expression patterns may be functionally related each other [26], we group the genes in the given data set according to functional similarity and define these gene clusters as *X*. The clustering results *C*s are then given by the proposed WDCM, k-means and SOM. We compute and compare the values of Adjusted Rand Index between *X *and *C*s to evaluate the performance of WDCM. To this end, we first use the Gene Functional Classification Tool of DAVID to group the genes into the highly functionally related gene clusters and then compute the values of *ARI*. The higher value indicates the corresponding clustering method performs better.

### Robustness of the WDCM on clustering incomplete data set

The WDCM can be applied to cluster the incomplete gene expression data set without imputing the missing values. To test the robustness of this approach, we compared the overlapped degree between the gene clusters for incomplete data sets and the ones for complete data sets. A higher overlapped degree represents a robust clustering method. To this end, we first randomly remove 5-25% of the complete data set in order to create the incomplete gene expression data sets, and then we apply the WDCM to cluster these complete and incomplete data sets and obtain the clustering results, respectively. Here, a Cluster Overlap Ratio (COR) index is introduced for assessing the overlapped degrees at individual missing percentages.

### Cluster Overlap Ratio index

Suppose *n *gene clusters *C*_1_,*C*_2_,...,*C*_*n *_for the complete data set and *m *gene clusters *I*_1_,*I*_2_, ... *I*_*m *_for the incomplete one. The Cluster Overlap Ratio (COR) index is then defined as follows:(5)

where(6)

|∙| denotes the number of genes in the cluster, and thus *p*_*i *_represents the proportion of genes in the gene cluster *I*_*i*_. Here *x*_*i *_denotes the maximum of overlapped gene numbers between *I*_*i *_and each individual *C*_*k *_(*k *= 1, ..., *n*) divided by |*I*_*i*_|.

## Results

### Identification of six gene clusters for lung cancer data set

**Figure 1:**
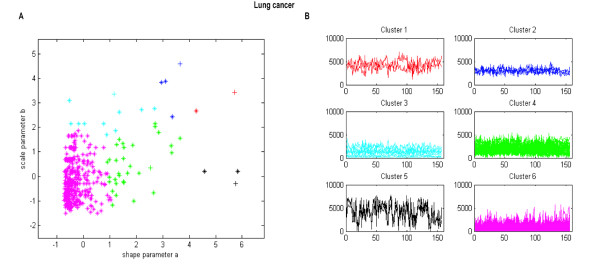
**Lung cancer data set clustered using the WDCM**. (A) Distribution parameters scatter plot. The horizontal axis corresponds to shape parameter a, and the vertical axis corresponds to scale parameter b. The parameter pairs in different clusters were drew with different colors. (B) Cluster profile plots.

We applied the WDCM to cluster the lung cancer data set. It consists of expression levels of 675 genes across 156 tissues, which include 17 normal and 139 carcinomas lung tissues [27]. Using the Kolmogorov-Smirnov goodness of fit test (see Methods), we tested whether the expression sample of each gene comes from the Weibull distribution. The results showed that the distributions of gene expressions of 402 genes belong to the common Weibull distribution, whereas the others whose distributions of gene expressions fail to be in the Weibull distribution are removed. The *p*-*values *produced by Kolmogoriv-Smirnov goodness of fit test for the 402 genes were reported in Additional file 2. We then used the hub node based clustering algorithm (see Methods) to cluster the 402 Weibull distribution parameters which consist of the shape parameters and scale parameters, and obtained 6 distribution parameter clusters, that is, 6 gene clusters. The clustered parameters scatter plots have been shown in Figure 1A.

It is evident from Figure 1A that the distribution parameters of the genes of a cluster are close and compact to each other, which indicates the Weibull distribution parameters were clustered well. The expression profiles of the corresponding clustered genes plots have been shown in Figure 1B, from which it is also evident that the expression profiles of the genes within identical clusters are quite similar, whereas the profiles for the genes belonging to different clusters differ from each other.

### Identification of four gene clusters for follicular lymphoma data set

**Figure 2:**
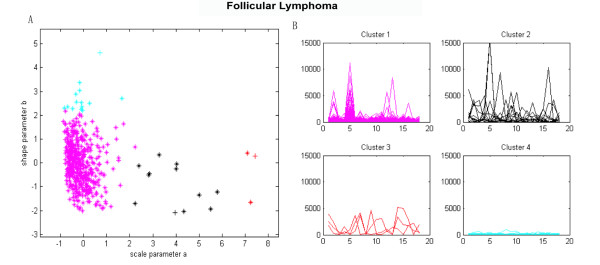
**Follicular lymphoma data set clustered using the WDCM**. (A) Distribution parameters scatter plot, (B) Cluster profile plots.

We tested the WDCM on another follicular lymphoma data set consisting of expression levels of 798 genes in 19 B-cell follicular lymphoma specimens [28]. We utilized the Kolmogorov-Smirnov test to decide if the sample of individual gene on the follicular lymphoma data set comes from the Weibull distribution, and found 471 genes whose distributions of gene expressions belong to the common Weibull distribution. The *p*-*values *produced by Kolmogoriv-Smirnov goodness of fit test for the 471 genes were reported in Additional file 2. We then clustered the corresponding 471 distribution parameter pairs and determined 4 gene clusters. Figure 2 illustrates the clustered parameters scatter plots and the cluster profile plots of the clustering results.

From Figure 2A, the four parameters clusters are clearly distinguished from each other, meanwhile, the expression profiles of the genes within the same clusters are similar, whereas the ones of the genes across different clusters are distinct (see Figure 2B). The results indicate that the significantly distinct gene clusters were found using the WDCM on follicular lymphoma data set.

### Identification of four gene clusters for bladder carcinoma data set

**Figure 3:**
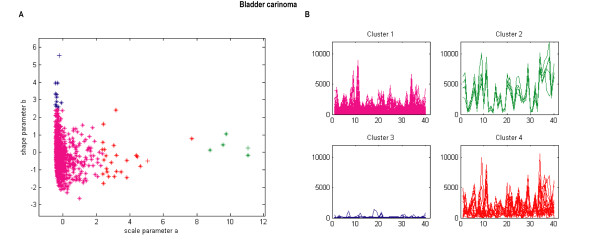
**Bladder carcinoma data set clustered using the WDCM**. (A) Distribution parameters scatter plot, (B) Cluster profile plots.

The bladder carcinoma data set contains 1203 genes measured over 40 different experimental conditions [29]. Using the Kolmogorov-Smirnov test, we found 1040 genes whose distributions of gene expressions belong to the common Weibull distribution. The *p-values *produced by Kolmogoriv-Smirnov goodness of fit test for the 1040 genes were reported in Additional file 2. Again, the hub node based clustering algorithm was employed to cluster the corresponding 1040 distribution parameter pairs. The number of clusters determined was 4. Figure 3 shows the clustered parameters scatter plots and the cluster profile plots of the clustering results.

### Comparison of clustering performance

To show the performance of the WDCM, we applied the K-means and Self Organizing Map (SOM) algorithms to the same gene subsets clustered by the WDCM and compared the functional consistency of the gene clusters produced by WDCM to those of the gene clusters produced by the other methods (see Methods). Simultaneously, the values of *ARI *for the WDCM, k-means and SOM algorithms on these three data sets were also contrasted (see Methods).

**Figure 4:**
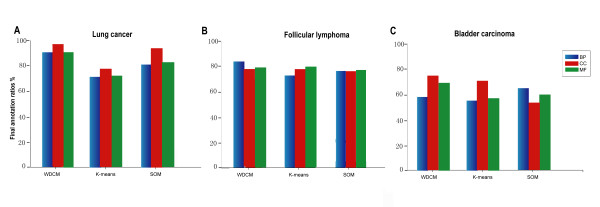
**biological annotation ratios of clustering results**. (A) Final annotation ratios of Lung cancer clusters found by three different methods in GO biological processes (BP), cellular components (CC) and molecular functions (MF). (B) Final annotation ratios of Follicular lymphoma clusters found by three different methods in GO biological processes (BP), cellular components (CC) and molecular functions (MF). (C) Final annotation ratios of Bladder carcinoma clusters.

Among these three tested algorithms, the WDCM show the highest functional annotation ratios on both lung cancer and follicular lymphoma data sets. The detailed comparisons for the lung cancer data set are given in Figure 4A, from which we found that the three final functional annotation ratios of the WDCM clusters all exceed the ones of the other methods clusters. Especially, the BP and MF annotation ratios of the WDCM clusters (91.57% and 92.16%) are much higher than those of the SOM clusters (82.76% and 83.96%). On B-cell follicular lymphoma data test, although the CC and MF annotation ratios of gene clusters found by each of three methods are asymptotically equal (see Figure 4B), the BP annotation ratio of WDCM clusters (84.9%) is much higher than those of K-means clusters (71.6%) and SOM clusters (74.8%). On bladder carcinoma data set, from Figure 4C, although the BP annotation ratio of WDCM clusters (59.82%) is less than those of SOM clusters (64.30%), it is still beyond that of K-means clusters (55.87%). Note that the CC and MF annotation ratios of the WDCM clusters are consistently superior to those of the K-means and SOM clusters.

Table 1 shows the values of *ARI *for algorithms WDCM, k-means and SOM on these three data sets. Note that among the three methods, WDCM provides the consistently best *ARI *values. Specifically, the *ARI *value for the proposed WDCM (0.5365) is much better than those for k-means and SOM (0.2478 and 0.3681) on lung cancer data set. Although these three *ARI *values (0.3991, 0.3481 and 0.2647) are close on B-cell follicular lymphoma data set, the *ARI *value for WDCM is better than the other values. For bladder carcinoma data set also, the proposed WDCM outperforms the other algorithms in terms of *ARI*. The values are reported in Table 1.

**Table 1:** *ARI *values of WDCM, k-means and SOM algorithms for the lung cancer, B-cell follicular lymphoma and bladder carcinoma gene expression data sets

Algorithm	Lung cancer	Follicular lymphoma	Bladder carcinoma
WDCM	**0.5365**	**0.3991**	**0.4105**
k-means	0.2478	0.3481	0.1623
SOM	0.3681	0.2647	0.0926

The above comparative analyses on the functional annotation ratios of the three algorithms have demonstrated that the genes in each cluster obtained using the WDCM show not only the similar expression patterns, but also more consistent functional annotations, which means these genes are more inclined to be involved in the same biological functions together. Also, the Adjusted Rand Index comparative results indicate the superiority of the performance of the proposed WDCM compared to the other algorithms.

### Test for robustness of the WDCM on clustering incomplete data set

To test the robustness with which the WDCM clusters the incomplete gene expression data, we applied the WDCM to cluster the above three gene expression data sets containing missing values and compared the overlapped degree between the gene clusters for incomplete data sets and the ones for complete data sets. These three data sets were preprocessed by randomly removing 5-25% of the data in order to create the incomplete gene expression data sets, and the WDCM then was applied to these data sets. Table 2 lists the average Cluster Overlap Ratio (COR) values with respect to the percentages of missing values (0-25%) achieved by WDCM over 100 runs for the lung cancer, B-cell follicular lymphoma and bladder carcinoma data sets, respectively. The WDCM provided the higher COR values regarding the smaller percentages of missing values for all three data sets. The COR values exceeded 0.9 at 5% missing value. At 10%, the COR value was also beyond 0.9 for the follicular lymphoma and bladder carcinoma data sets (0.9078 and 0.9702), and approximated 0.9 for the lung cancer data set (0.8654). For the bladder carcinoma data set, we see that the COR values were varied from 0.9823 to 0.9335, passing 0.9 at all missing values.

**Table 2:** COR indices with respect to the specified percentages of missing values for the lung cancer, B-cell follicular lymphoma and bladder carcinoma data sets

Percentage of missing	Lung cancer	Follicular lymphoma	Bladder carcinoma
5%	0.9140	0.9495	0.9823
10%	0.8654	0.9078	0.9702
15%	0.8220	0.8738	0.9565
20%	0.7892	0.8418	0.9450
25%	0.7649	0.8120	0.9335

The results of the cluster overlapped degree comparison tests indicate that the WDCM gave a high overlapped degree of the gene clusters compared with those of complete data set at low missing value, highlighting the robustness and potential of the WDCM. We think that the results might stem from the fact that the missing gene expression values of individual genes have little influence on constructing their corresponding Weibull distribution parameters at low missing values.

## Discussion and conclusion

In this article, we propose a robust approach based on Weibull distribution (WDCM) for clustering gene expression data. It is based on the idea that a group of genes tend to be clustered together if the distributions of gene expressions of these genes belong to the common Weibull distribution and have the similar distribution parameters. Consequently, we cluster the genes by clustering the distribution parameters of their gene expressions. A hub nodes-based dynamic clustering algorithm is utilized in the distributions clustering process. The clusters number in a gene expression data set is automatically determined in this clustering algorithm. The performance of the proposed WDCM has been compared with those of K-means and SOM clustering algorithms by the biological annotation ratios to show its effectiveness on three cancer gene expression data sets. The results show that the WDCM is more capable of grouping the genes with similar expression patterns and strong functional consistency together. We also used the external measure Adjusted Rand Index to validate the performance of the WDCM. The comparative results demonstrate that the WDCM provides the better clustering performance compared to k-means and SOM algorithms. Moreover, the WDCM can be applied to cluster the incomplete gene expression data set without imputing the missing values. The results have demonstrated that there is high overlap between the gene clusters for the incomplete data set and those for the complete data set, which illustrates the robustness of the WDCM on clustering the incomplete data set at low percentage of missing values.

In general it is known that due to the complex nature of the gene expression data sets themselves and the experimental errors in detecting the gene expression data, it is difficult to discover an acknowledged best clustering approach. In clustering process, the WDCM disregards a few genes whose gene expression distributions fail to fit the Weibull distribution. In future study, we will consider replacing the single Weibull distribution with the mixture distribution in order to cluster the whole data set. Besides, we will also increase the robustness of this approach on clustering the incomplete gene expression data set containing the missing values of moderate percentage. For the gene clusters found by WDCM, we would like to investigate which gene clusters and genes are correlated with some cancer phenotype, and which biological processes or molecular functions these genes in the clusters are concerned with. Our study may be helpful to gain insights into the complex diseases.

## Competing interests

The authors declare that they have no competing interests.

## Authors' contributions

HKW and ZZW jointly proposed this approach and conducted the data experiments. XL gave the statistical idea of the method. BSG modified this paper. LXF partly wrote the program codes. Testing was done by YZ. All authors read and approved the final manuscript.

## Supplementary Material

Additional file 1 **A clustering algorithm based on "hub nodes"**. A clustering algorithm used to cluster the Weibull distribution parameters. Click here for file

Additional file 2 **P-values of tests for the three data sets**. This file consists of three spreadsheets, each lists the gene numbers and p-values of Kolmogorov Smirnov test for one data set. Click here for file
