# Supplementary material for: A robust approach based on Weibull distribution for clustering gene expression data
Source: Algorithms Mol Biol. 2011 May 31;6:14. doi: 10.1186/1748-7188-6-14 (PMC3118357; doi:10.1186/1748-7188-6-14)
Supplement: Additional file 1 — A clustering algorithm based on "hub nodes". A clustering algorithm used to cluster the Weibull distribution parameters. [file 1748-7188-6-14-S1.PDF]

A clustering algorithm based on "hub nodes"

Step 1. From n parameter pairs $(a_1, b_1), \cdots, (a_n, b_n)$, choose any one to be the first hub, and denote it as hub1.

Step 2. Define the distance between the arbitrary two points (a_i, b_i), (a_j, b_j)

$d_{i,j} = \sqrt{(a_i - a_j)^2 + (b_i - b_j)^2}$, $i, j = 1, 2, \cdots, n$. Find the point which is farthest from hub1, and make it the second hub, denote it as hub2.

Step 3. For each remaining point, calculate the distance from this point to each hub and assign it to the hub to which it is closer.

Step 4. To determine whether it is necessary to find a third hub, do the following:

4.1 Define the stop value $R = \frac{d_{hub1,hub2}}{2}$.

4.2 Calculate the distance from each point to its hub. If there is any distance which is greater than R, it is necessary to find a third hub.

Step 5. To find a third hub, find the point which is farthest from its hub and make it hub3.

Step 6. Calculate the distance from each point to hub3 to see if that distance is less than the point's distance to its current hub. If so, reassign this point to hub3.

Step 7. Let $R = \frac{1}{2} \sum_{i,j=1}^{m} \frac{d_{hubi,hubj}}{m}$, here m is the number of the hubs.

Step 8. Once again, see if any distance from a point to its hub exceeds R .

Step 9. Continue this process until all points' distances to their current hubs are within R or until all points themselves hubs.
